# Mitochondrial Membranes and Mitochondrial Genome: Interactions and Clinical Syndromes

**DOI:** 10.3390/membranes12060625

**Published:** 2022-06-15

**Authors:** Mohammed Almannai, Azza Salah, Ayman W. El-Hattab

**Affiliations:** 1 Genetics and Precision Medicine Department, King Abdullah Specialized Children Hospital, Riyadh P.O. Box 22490, Saudi Arabia; 2 Department of Pediatrics, University Hospital Sharjah, Sharjah P.O. Box 72772, United Arab Emirates; azza.slh@gmail.com; 3 Department of Clinical Sciences, College of Medicine, University of Sharjah, Sharjah P.O. Box 27272, United Arab Emirates; elhattabaw@yahoo.com; 4 Genetics and Metabolic Department, KidsHeart Medical Center, Abu Dhabi P.O. Box 505193, United Arab Emirates

**Keywords:** mitochondria, IMM, OMM, fission, fusion, mtDNA

## Abstract

Mitochondria are surrounded by two membranes; the outer mitochondrial membrane and the inner mitochondrial membrane. They are unique organelles since they have their own DNA, the mitochondrial DNA (mtDNA), which is replicated continuously. Mitochondrial membranes have direct interaction with mtDNA and are therefore involved in organization of the mitochondrial genome. They also play essential roles in mitochondrial dynamics and the supply of nucleotides for mtDNA synthesis. In this review, we will discuss how the mitochondrial membranes interact with mtDNA and how this interaction is essential for mtDNA maintenance. We will review different mtDNA maintenance disorders that result from defects in this crucial interaction. Finally, we will review therapeutic approaches relevant to defects in mitochondrial membranes.

## 1. Introduction

Mitochondria are ubiquitous organelles that are essential in cellular-energy production [1]. They also play key roles in several other biological processes such as cellular metabolism, calcium homeostasis, and apoptosis [1,2]. Mitochondria are surrounded by two membranes: the outer mitochondrial membrane (OMM) and the inner mitochondrial membrane (IMM). The outer membrane has pore-forming membrane proteins (porins) that allow for free transport of ions and small, uncharged molecules [3]. On the other hand, the inner membrane is impermeable, and this feature is essential to maintain electrical-membrane potential that drives adenosine-triphosphate (ATP) synthesis. Molecules are therefore transported across IMM through specific transporters [4]. Electron-transport-chain (ETC) complexes are embedded in the IMM, where energy production through oxidative phosphorylation takes place. To optimize this function, the IMM is folded into structures called “cristae”, which significantly increases the surface area of the IMM [5].

Unlike other organelles, the mitochondria contain their own DNA, the mitochondrial DNA (mtDNA). MtDNA is maternally inherited [6] and each cell contains several hundreds to thousands of copies of mtDNA, depending on the cell type [7]. MtDNA is maintained by a group of nuclear-encoded proteins that are involved in mtDNA replication and repair. These nuclear-encoded proteins are either components of the mtDNA replisome itself or perform other functions essential for mtDNA synthesis, such as supply of deoxynucleotide triphosphates (dNTPs) and mediating mitochondrial fission and fusion [8]. Mitochondrial membranes have direct interaction with mtDNA, and are therefore involved in mtDNA maintenance in several aspects, including organizing the mitochondrial genome, facilitating mitochondrial dynamics, and controlling the mitochondrial nucleotides pool [9,10,11].

Mitochondrial DNA maintenance defects represent a growing list of disorders caused by defects in the nuclear-encoded proteins involved in mtDNA maintenance, resulting in impaired mtDNA synthesis with quantitative (mtDNA depletion) and/or qualitative (multiple mtDNA deletions) defects in mtDNA. These conditions have multisystem involvement with significant morbidity and mortality [8]. Therapeutics options in these disorders remain limited, and therefore, understanding the underling pathophysiological mechanisms in these disorders is of prime importance to build more specific and efficient therapies.

In this review, we will discuss the mechanisms of mtDNA synthesis and the interaction of mitochondrial membranes with mtDNA and how this interaction is essential for mtDNA maintenance. Then, we will review primary mtDNA maintenance disorders that result from defects in this crucial interaction, including defects in nucleotide organization (*TFAM*, *TWNK*, and *ATAD3A*-related disorders), disorders of mitochondrial fission and fusion (*DNM1L*, *MFF*, *OPA1*, and *MFN2*-related disorders), cardiolipin-related disorders (Barth syndrome), and finally, disorders related to mitochondrial membrane transporters (*MPV17* and *ANT*-related disorders) (Table 1).

## 2. Mitochondrial DNA Synthesis

MtDNA is a double-stranded circular DNA around 16.6 Kb in length that contains 37 genes; 13 of which encode subunits of the ETC, while the remaining 24 genes encode for 22 transfer RNAs (tRNA) and two ribosomal RNAs (rRNA) [12,13]. The two strands of mtDNA are denoted as the ‘heavy’ (H) and ‘light’ (L) strands. Besides several short intergenic noncoding regions, the mtDNA contains a large noncoding D-loop, which is a triple-stranded region that acts as a promoter for the heavy and light strands and contains essential transcription and replication elements [14]. Replication of mtDNA is a continuous process, independent of cell cycle, and it requires many nuclear-encoded proteins that are either components of the mtDNA replisome itself or perform other functions essential for mtDNA synthesis such as the supply of dNTPs and mediating mitochondrial fission and fusion [8].

**Figure 1:**
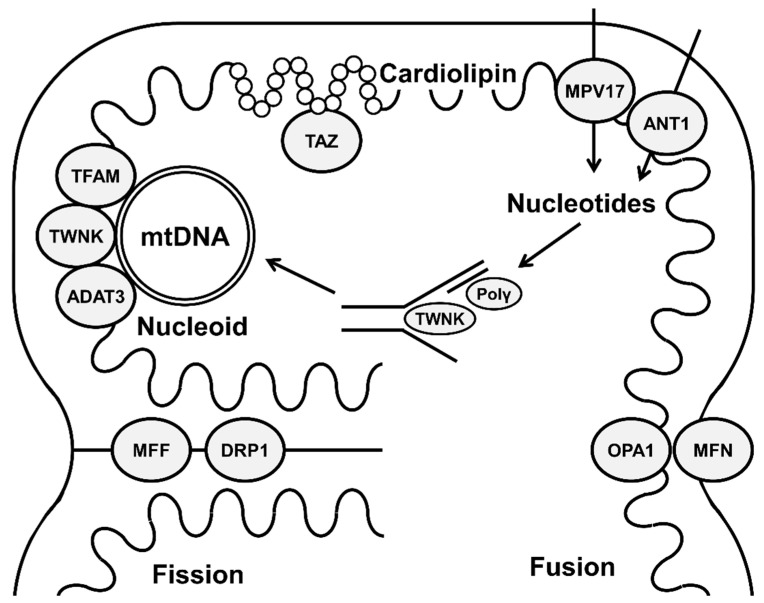
Schematic representation showing different mitochondrial-membrane proteins involved in mitochondrial-genome organization and maintenance.

MtDNA replication involves two asynchronous unidirectional origins of replication [15]. It is replicated by polymerase gamma (polγ), a heterotrimer consisting of a catalytic subunit encoded by *POLG* and a homodimer of two accessory subunits encoded by *POLG2* [16]. Other components of mtDNA replisome include TWINKLE (the mitochondrial DNA helicase) [17], mitochondrial topoisomerase I, and mitochondrial single-stranded DNA-binding protein [18] (Figure 1).

Compared to nuclear DNA repair, mtDNA repair mechanisms are not completely understood, with the most characterized mechanism being base excision repair (BER) [19]. Although proteins involved in mismatch repair (MMR) and DNA double-strand break (DSB) mechanisms have been found in mitochondria, it is still debated whether these mechanisms are involved in mtDNA repair [19]. The mechanism of nuclear and mitochondrial BER are similar and involve recognition and removal of the modified base and then incorporation of the correct nucleotide and strand ligation [20]. Besides mtDNA repair, damaged mtDNA molecules can be degraded, therefore reducing the population of damaged mtDNA, and then replication of the intact mtDNA can maintain adequate mtDNA copies [19].

## 3. Mitochondrial Membranes and the Organization of the Mitochondrial Genome

There is a network of interaction between mtDNA, different proteins, and mitochondrial membranes that organize the mitochondrial genome in a way to make the continuous replicative nature of mtDNA efficient. This delicate organization helps the mitochondria to respond to cellular metabolic needs. Several mitochondrial-membrane proteins play essential roles in mitochondrial-genome organization. These proteins are encoded by nuclear genes in which pathogenic variants cause variable primary mtDNA maintenance defects including *TFAM*, *TWNK*, and *ATAD3A*-related disorders. These conditions are often associated with early-onset, severe, multiorgan diseases with prominent neurological manifestations, indicating the vital roles these proteins play in maintaining integrated mtDNA, which is essential for mitochondrial function.

### 3.1. Molecular Basis of Mitochondrial Genome Organization

The mitochondrial genome is located in the mitochondrial matrix, the interior space enclosed by the IMM. MtDNA is packaged into structures called nucleoids, spherical structures that frequently contain a single copy of mtDNA (average ∼1.4 mtDNA molecules per nucleoid) [21]. MtDNA is packed with a number of nucleoid-related proteins, including proteins of the mtDNA replication and transcription [10,22]. Nucleoids are dynamic structures that distribute evenly throughout the mitochondrial matrix and their distribution depends on continuous mitochondrial fusion and fission [23]. Moreover, the D-loop region of mtDNA is anchored to the IMM through a multiprotein complex [10]. The attachment of nucleoids to the IMM is essential to ensure that the mitochondrial genome is distributed throughout the mitochondria [24].

The mitochondria are physically connected to endoplasmic reticulum (ER) through what are called mitochondrial-associated ER membranes (MAMs). MAMs consist of proteins and lipids located in the OMM and ER membrane and are essential for mitochondrial fission and fusion [25]. Moreover, mtDNA synthesis within nucleoids is spatially linked to MAMs. The mitochondrial–ER contact could mediate initiation of mtDNA transcription and then distribution of newly replicated nucleoids to daughter mitochondria [26]. Using live-cell super-resolution imaging, it was shown that the ER–mitochondria contact sites are essential for nucleoids dynamics, segregation, and transportation [27].

One of the core components of mitochondrial nucleoids is mitochondrial transcription factor A (TFAM), which is an IMM protein. TFAM is a master transcription factor in the mitochondria, but it also plays important roles in coating and packaging the mitochondrial genome [28]. The degree of nucleoid compaction is affected by TFAM protein levels, which is an important mechanism for control of mtDNA expression [23]. TFAM regulates nucleoid structure, and knockdown of TFAM was associated with mtDNA depletion and severe nucleoid aggregation [29]. Twinkle, the mtDNA helicase, is another component of the nucleoids and it is firmly associated with IMM, even in the absence of mtDNA, indicating that Twinkle recruits or is assembled with mtDNA at the IMM to form a replication platform [30]. In *Drosophila melanogaster*, it was shown that binding of TWINKLE to IMM is achieved through iron-sulfur clusters, and membrane binding was more specific with increasing cardiolipin content [31]. This interaction with the IMM may serve to recruit mtDNA and initiate mtDNA unwinding and replication [31].

ATAD3 (ATPase Family AAA Domain Containing 3), an IMM protein, has been linked to nucleoid organization [32]. The ATAD3 cluster is composed of three paralogs with sequence homology: ATAD3A, ATAD3B, and ATAD3C. ATAD3A and ATAD3B are protein-coding genes, whereas ATAD3C is not [33]. Using neuronal-specific conditional Atad3 knockout mouse model, Arguello et al. showed the fundamental structural role of ADAT3A in organization of IMM [34]. In HeLa cells, the authors showed that most mtDNA is associated with ATAD3A, but not all ATAD3A foci are associated with mtDNA nucleoids, suggesting a compartmentalization role for ATAD3A [34].

### 3.2. Clinical Syndromes due to Defects in Mitochondrial Genome Organization

A homozygous missense pathogenic variant in *TFAM* was identified in two siblings who presented a fatal phenotype characterized by intrauterine growth retardation, liver failure, and death in early infancy. MtDNA content was reduced in liver and muscle [35]. In a recent report, three affected individuals from one family with a homozygous missense variant in *TFAM* presented with seizures and intellectual disability. The affected females developed primary ovarian insufficiency. Fibroblasts from affected individuals showed mtDNA depletion with altered mitochondrial function and morphology. Interestingly, abnormal nucleoids were identified with reduced nucleoid numbers and significant changes in nucleoid size or shape, indicating the important role of TFAM in nucleoids organization [36].

Monoallelic pathogenic variants in *TWNK* gene cause adult-onset autosomal dominant progressive external ophthalmalgia (PEO), which present with PEO, ptosis, and variable other features such as myopathy [37]. Biallelic pathogenic variants in *TWNK* cause autosomal recessive infantile-onset spinocerebellar ataxia (IOSCA) and Perrault syndrome. IOSCA is a severe disease characterized by normal development for one year followed by progressive hypotonia, ataxia, athetosis, ophthalmoplegia, and hearing loss. Other features include sensory axonal neuropathy, epilepsy, and hypergonadotropic hypogonadism in females [38]^.^ Hakonen et al. showed that IOSCA was associated with tissue-specific mtDNA depletion in the brain and the liver. Nucleoid structure was normal [39]. Perrault syndrome is associated with sensorineural hearing loss and ovarian dysfunction [40].

Dominant and recessive pathogenic variants in *ATAD3A* have been recently linked to human disease. In 2016, Harel et al. reported five unrelated individuals with a recurrent, *de novo* pathogenic variant in *ATAD3A* (c.1582C>T; p.Arg528Trp) [41]. These individuals presented with global developmental delay, hypotonia, optic atrophy, axonal neuropathy, and hypertrophic cardiomyopathy. In the same report, two additional families with biallelic variants in *ATAD3A* were also reported. Fibroblasts of an affected individual showed increased mitophagy [41]. Skeletal-muscle-specific *atad3a* knockout mice developed progressive mtDNA depletion and deletions [42]. Desai et al. identified four individuals with a fatal pontocerebellar hypoplasia and respiratory insufficiency syndrome [43]. All died within the first week of life and were found to have biallelic deletions within the *ATAD3* gene cluster on chromosome 1p36.33 and these deletions created an *ATAD3B*/*ATAD3A* fusion gene. In the same report, Desai et al. also reported a fifth individual with later-onset encephalopathy with cerebellar atrophy who had genomic rearrangements affecting the *ATAD3C*/*ATAD3B* genes on one allele and *ATAD3B*/*ATAD3A* genes on the other [43]. Affected individuals’ fibroblasts showed enlarged mtDNA foci, suggesting ATAD3 deficiency causes impaired distribution and localized aggregation of mtDNA. ATAD3 deficiency was also associated with slow mtDNA synthesis [43]. A monoallelic reciprocal duplication at the ATAD3 locus was identified in five neonates who presented with a lethal phenotype characterized by cardiomyopathy, corneal opacities, encephalopathy, hypotonia, and seizures [44]. The duplication produces a fusion gene derived from *ATAD3A* and *ATAD3C*, the protein product of which lacks key functional residues. Fibroblasts from two individuals show evidence of abnormal mitochondrial morphology, mtDNA organization, and cholesterol metabolism [44].

## 4. Mitochondrial Fission and Fusion and Maintaining Mitochondrial Genome

Mitochondria constantly fuse and divide [45]. This dynamics nature requires interaction of several OMM and IMM proteins. Through continuous fission and fission, the mitochondrial proteome is well-balanced and this feature, as we will discuss below, is essential for mtDNA synthesis. The role of mitochondrial dynamics in mtDNA synthesis is further supported by the fact that several disorders of mitochondrial fusion and fission are associated with defects in mtDNA (*DNM1L*, *MFF*, *OPA1*, and *MFN2*-related disorders). The understanding of the delicate interaction of mitochondrial dynamics and mtDNA maintenance will help towards making targeted therapeutic agents in these disorders (see Section 7).

### 4.1. Molecular Basis of Mitochondrial Fission and Fusion

Mitochondrial fission is the division of mitochondria into multiple new mitochondria. It is achieved through progressive constriction of the IMM and OMM and then scission of the membrane through action of dynamin-related protein 1 (DRP1), which interacts with several adaptor proteins on the OMM [11]. Mitochondrial fission factor (MFF) is an OMM protein that is essential for fission and it is one of the major DRP1 receptors [46]. Mitochondrial fusion, the physical merging of two mitochondria, is mediated through action of three GTPase. Mitofusin 1 (MFN1) and mitofusin 2 (MFN2) mediate fusion of the OMM and the dynamin-related protein OPA1 is essential for IMM fusion [9,47].

MtDNA integrity is affected by alterations in protein stoichiometry [9]. In mouse embryonic fibroblasts and mouse cardiomyocytes, loss of OMM fusion resulted in nucleoid clustering. The mouse hearts lacking OMM fusion showed profound mtDNA depletion that resulted from alterations in stoichiometry of the protein components of the mtDNA replisome [48].

### 4.2. Clinical Syndromes due to Defects in Mitochondrial Fission

Monoallelic and biallelic pathogenic variants in *DNM1L*, which encodes DRP1, causes encephalopathy due to defective mitochondrial and peroxisomal fission 1 (EMPF1). This disorder is characterized by early-onset hypotonia, severe developmental delay, microcephaly, optic atrophy, and failure to thrive [49,50]. Electron microscopy showed giant mitochondria with elongated cristae [49]. Besides this severe phenotype, Gerber et al. identified a dominant form of *DNM1L*-related disorder in three families with isolated optic atrophy [51]. Biallelic pathogenic variants in *MFF* cause encephalopathy due to defective mitochondrial and peroxisomal fission 2 (EMPF2), a very rare disease associated with developmental delay, severe hypotonia, and abnormal signals in the basal ganglia [52]. As expected, given the role of MFF in mitochondrial (and peroxisomal) fission, affected individuals’ fibroblasts showed elongated mitochondria and peroxisomes with increased mitochondrial branching [53].

### 4.3. Clinical Syndromes due to Defects in Mitochondrial Fusion

Monoallelic pathogenic variants in *OPA1* are associated with autosomal dominant optic atrophy, which could be isolated or associated with other symptoms “Optic atrophy ‘plus’ phenotype”. The later is associated with sensorineural hearing loss, progressive external ophthalmoplegia, ataxia, and neuropathy. Multiple mtDNA deletions are evident in skeletal muscle [54]. Biallelic pathogenic variants in *OPA1* are associated with Behr syndrome, an autosomal recessive disorder characterized by early-onset optic atrophy along with ataxia, pyramidal signs, and intellectual disability [55]. Finally, one case report described two sisters with a homozygous pathogenic variant in *OPA1* who presented with a fatal encephalomyopathy phenotype associated with severe mtDNA depletion in skeletal muscle [56].

Monoallelic pathogenic variants *MFN2* cause optic atrophy ‘plus’ phenotype [57]. Pathogenic variants in this gene are also associated with dominant and recessive forms of Charcot–Marie–Tooth disease type 2A (CMT2A) [58]. Compared to CMT1A, which is the commonest form of CMT, CMT2A is associated with a more severe, and a motor-predominant phenotype that manifests earlier in life. Additional features that could develop include optic atrophy, vocal-cord involvement, upper motor-neuron dysfunction, and white-matter lesions on brain MRI [59].

## 5. Cardiolipin and Mitochondrial-Genome Stability

The major classes of phospholipids in the mitochondrial membrane are similar to other membranes with phosphatidylcholine and phosphatidylethanolamine being the most abundant phospholipids. Phosphatidylglycerol and cardiolipin are exclusive components of the mitochondrial membranes, with the latter making approximately 15% of total phospholipids. The mitochondrial membranes also have low levels of sphingolipids and cholesterol [60]. Cardiolipin is a hallmark lipid of mitochondria and is predominantly located in the IMM, where it has been linked to several physiological functions [61]. Cardiolipin has a unique dimeric structure with four acyl chains and two phosphatidyl moieties linked by a glycerol bridge [62]. It is essential to maintain the shape and stability of mitochondrial cristae [63]. Cardiolipin is also involved in oxidative phosphorylation and energy production. It closely interacts with ETC, contributing to the organization of ETC complexes into supercomplexes for more efficient energy production [64]. Cardiolipin is essential for mtDNA maintenance. Barth syndrome is a primary mitochondrial disease resulting from deficiency of tafazzin, which is essential for cardiolipin remodeling.

### 5.1. Role of Cardiolipin in mtDNA Maintenance

Cardiolipin is essential for mitochondrial dynamics, including fission, fusion, and mitophagy [65]. Cardiolipin is involved in mitochondrial fusion through biogenesis and assembly of OPA1 [66]. It directly interacts with OPA1 to promote membrane fusion [67]. Likewise, cardiolipin interacts with DRP1, working cooperatively to achieve membrane constriction and eventually fission during mitochondrial division [68]. Furthermore, cardiolipin binds to nucleoids and is essential for mtDNA stability and segregation. Luevano-Martinez et al. showed that mtDNA physically interacts with cardiolipin both in yeast and in mammalian mitochondria. Cardiolipin synthase-null mutant strain was not able to adapt to thermal stress, as lack of cardiolipin makes mtDNA more prone to damage under such stress conditions. This protective effect of cardiolipin under stress was linked to its role in binding to mitochondrial nucleoids and guiding their segregation between cells [69].

Cardiolipin is synthesized through condensation of phosphatidylglycerol and cytidinediphosphate-diacylglycerol (CDP-DAG) at the IMM through action of cardiolipin synthase [70]. Cardiolipin is then remodeled through cycles of deacylation and reacylation to achieve the specific fatty acyl composition unique for cardiolipin [71].

### 5.2. Clinical Syndromes due to Defects in Cardiolipin Metabolism

Cardiolipin transacylase (tafazzin), encoded by *TAFAZZIN* gene, is an enzyme involved in remodeling monolysocardiolipin to mature cardiolipin [72]. Barth syndrome is a rare, X-linked mitochondrial disease resulting from a pathogenic variant in *TAFAZZIN*. Barth syndrome is characterized by the triad of mitochondrial myopathy, neutropenia, and cardiomyopathy. Cardiomyopathy is usually dilated. Left-ventricular noncompaction is also commonly seen. There are increased urinary levels of 3-methylglutaconic acid. Additional features include exercise intolerance, lactic acidosis, and growth delay [73]. Electron-microscopy findings include enlarged mitochondria and abnormal mitochondrial cristae [74]. In one report, mtDNA content in fibroblasts from an affected individual with a frameshift variant in *TAFAZZIN* showed significant decrease in mtDNA copy numbers compared to healthy controls, whereas the cell line with a missense variant did not show a similar decrease. Nevertheless, both cell lines showed a significant increase in mtDNA copy numbers following adeno-associated virus (AAV) gene delivery [75].

## 6. Mitochondrial-Membrane Transporters and mtDNA Maintenance

Transport of molecules is tightly regulated at the level of IMM. As the mitochondria require constant supply of nucleotides for mtDNA replication, there must be a crucial role for IMM transporters in such process. Defects in proteins such as MPV17 and ANT1 have been associated with disorders of mtDNA maintenance as discussed below. Although the exact role of these proteins may not be completely understood, they are probably involved in maintaining an adequate nucleotide pool for mtDNA replication.

### 6.1. Role of Mitochondrial Membrane Transporters in mtDNA Maintenance

MtDNA synthesis requires a constant and balanced supply of dNTPs. Synthesis of dNTPs is archived through the *de novo* and salvage pathway in the cytosol, whereas inside the mitochondrial matrix, only the salvage pathway operates, and it requires two enzymes: thymidine kinase 2 (TK2) and deoxyguanosine kinase (DGK) [76]. In proliferating cells, the mitochondrial dNTPs are mainly imported from the cytosol [76].

As mentioned before, the IMM impermeable, and therefore molecules, including nucleotides, are transported through specific transporters known as the mitochondrial carrier family and encoded by *SLC25* genes [77]. These include the human equilibrative nucleoside transporter (ENT) family, which transport both purines and pyrimidines [78] and pyrimidine nucleotide carriers (PNC) [79].

Defects in two IMM transports, MPV17 and ANT1 (adenine nucleotide translocator 1), cause different forms of mtDNA maintenance defects. While the exact role of these proteins in mtDNA maintenance is not completely understood, they are probably involved in maintaining a balanced mitochondrial dNTPS pool.

The exact function of MPV17 is not completely understood. Electrophysiological studies showed that MPV17 is a nonselective channel with gating regulated by different factors including membrane potential, redox state, and protein phosphorylation [80]. Using nuclear magnetic resonance (NMR), it was shown that MPV17 protein has six membrane-embedded α-helices [81]. Under oxidative stress, it forms disulfide-stabilized oligomeric pores and these may serve to transport DNA precursors into the mitochondrial matrix to compensate for damage caused by oxidative stress. Pathogenic variants in *MPV17* prevent these oligomerization properties [81].

The association of defects in MPV17 with mtDNA depletion suggest a role of MPV17 in mtDNA maintenance and dNTPs pools. In the knockout mice model, Dalla Rosa et al. showed that *Mpv17* ablation resulted in a decrease in the levels of deoxyguanosine triphosphate(dGTP) and deoxythymidine triphosphate (dTTP) in liver mitochondria along with severe mitochondrial DNA depletion, whereas these changes were not observed in brain and kidney mitochondria [82]. Fibroblasts from affected individuals also showed decreased dNTPs levels and mtDNA depletion, and this was rescued by deoxynucleoside supplementation [82]. In *mpv17*^−/−^ zebrafish model, treatment with pyrimidine precursor orotic acid resulted in significant increase in mtDNA content [83]. In HeLa cells, decreased MPV17 expression was associated with abnormal folate metabolism, indicating impaired deoxythymidine monophosphate (dTMP) synthesis. MPV17 may therefore be involved in the transport of dTMP from the cytosol to the mitochondria for mtDNA synthesis [84].

The mitochondrial ADP/ATP carrier (adenine nucleotide translocator, ANT) is encoded by four genes that have different tissue expression: *SLC25A4* (ANT1), *SLC25A5* (ANT2), *SLC25A6* (ANT3), and *SLC25A31* (ANT4). While ANT3 is ubiquitously expressed, ANT1 is highly expressed in the heart, skeletal muscle, brain, and organs with low mitotic regeneration, and ANT2 is more expressed in proliferating tissues [85]. ANT4 was recently identified and specifically expressed in the testes [86].

ANTs are the most abundant proteins in the IMM where they are bound to membrane lipids including cardiolipin and phosphatidic acid [87]. ANTs catalyze the exchange of ATP generated in mitochondria with ADP [88]. Through this exchange, ANTs have a vital role in energy metabolism by maintaining high ATP concentrations in the cytosol for different energy-requiring reactions [89]. ANTs may have a role in mitochondrial permeability transition [90]. Finally, given the defects in mtDNA maintenance in *SLC25A4*-associated disorders, it is postulated also that ANTs could be involved in maintaining a balanced nucleotide pool in the mitochondrial matrix [91].

### 6.2. Clinical Syndromes due to Defects in Mitochondrial-Membrane Transporters

In 2006, Spinazzola et al. identified variants in the mouse kidney-disease gene (*MPV17*) in three families with hepatocerebral mtDNA maintenance defect, and showed that MPV17 is a IMM protein [92]. Since then, several cases have been reported with biallelic pathogenic variants in *MPV17,* with two main phenotypes: an early-onset encephalohepatopathic disease characterized by liver dysfunction that typically progresses to liver failure, neurological manifestations (developmental delay, hypotonia, and peripheral neuropathy), lactic acidemia, and mtDNA depletion detected mainly in liver tissue, but can be also detected in skeletal muscles; and less commonly, MPV17 deficiency can cause a late-onset disease characterized by myopathy and peripheral neuropathy with minimal or no liver involvement [93].

Human diseases have been also associated with defects in ANT1, encoded by *SLC25A4.* Monoallelic pathogenic variants in *SLC25A4* cause adult-onset progressive external ophthalmoplegia that could be associated with exercise intolerance, muscle weakness, sensorineural hearing impairment, psychiatric illnesses, and multiple mtDNA deletions [94]. Biallelic pathogenic variants in *SLC25A4* are associated with a hypertrophic cardiomyopathy phenotype that presents early in childhood and is associated with lactic acidosis, myopathy, and cataracts, as well as multiple mtDNA deletions in skeletal muscle [95]. Interestingly, loss of acylglycerol kinase (AGK), an enzyme that catalyzes the phosphorylation of diacylglycerol and monoacylglycerol to phosphatidic acid and lysophosphatidic acid, results in a decrease in ANT1 in the IMM in muscle, suggesting a role for AGK in in the assembly and stabilization of the ANTs [96]. AGK deficiency causes Sengers syndrome; an autosomal recessive disease characterized by congenital cataract, hypertrophic cardiomyopathy, skeletal myopathy, lactic acidosis, and mtDNA depletion [97].

## 7. Therapeutic Strategies

Understanding how the mitochondrial membranes interact with the mitochondrial genome and support mtDNA synthesis can open venues for specific and efficient therapeutic options to treat mtDNA maintenance defects. Treatment of such disorders have been mostly supportive and symptom-specific, but fortunately the number of preclinical and clinical trials have grown significantly recently [98].

One of the therapeutic approaches tried for *OPA1*-related mitochondrial diseases is genome editing using small ribonucleoprotein particle U1 (U1 snRNP), especially since 30% of *OPA1* pathogenic variants are splice-site [99]. Jüschke et al. showed that engineered U1 splice factors targeted to intron 10 can ameliorate the effect of c.1065+5G>A variant, which causes exon 10 skipping, and this proof-of-concept study indicates the feasibility of splice-site variant correction as a therapeutic option for *OPA1*-related mitochondrial diseases [99]. Another therapeutic approach that is being evaluated in individuals with dominant optic atrophy (DOA), including those with *OPA1* pathogenic variants, is autologous bone-marrow-derived stem cells (BMSC). An open label, nonrandomized study showed that BMSC can result in significant visual improvements and this study is still ongoing and recruiting subjects [100] https://clinicaltrials.gov/ct2/show/NCT03011541, accessed on 8 May 2022). Gene therapy using viral and nonviral vectors has also been tried in animal models [101,102].

As discussed before, MFN2 is involved in fusion of the OMM. It was shown that interactions between specific amino-acid residues within MFN2 can change protein conformation, which in turn has an impact on the function of MFN2 in mediating membrane fusion [103]. The closed conformation, which is stabilized by the alignment of specific amino-acid residues in the heptad repeat (HR) 1 and 2 domains, is fusion-incompetent. Therefore, molecules that disrupt the interactions that maintain the closed conformation can promote mitochondrial fusion [104]. Rocha et al. showed that these so-called “mitofusin agonists” reversed mitochondrial fragmentation, dysmotility, and hypopolarization in cultured mouse neurons expressing mutants MFN2 [104]. Recently, a 4-hydroxy cyclohexyl analog was suggested to be a good candidate as a “mitofusion agonist” for preclinical studies [105].

Another therapeutic approach explored in recent studies is cardiolipin stabilization. Szeto–Schiller (SS) peptides are molecules that selectively target the IMM [106]. One of them is SS-31 (Elamipretide), which binds selectively to cardiolipin via electrostatic and hydrophobic interactions. It can maintain mitochondrial cristae, therefore promoting oxidative phosphorylation and reducing oxidative stress [107]. A phase 3 trial to evaluate the efficacy of elamipretide in subjects with primary mitochondrial myopathy (MMPOWER-3) was recently terminated as it did not meet its primary endpoints (https://clinicaltrials.gov/ct2/show/NCT03323749, accessed on 8 May 2022). It was noted, though, that there was an improvement in a subset of subjects with disorders affecting the mtDNA replisome. Based on this observation, there is currently an active and recruiting trial to evaluate the efficacy and safety of elamipretide in subjects with primary mitochondrial disease resulting from nuclear DNA mutations (NuPower) (https://clinicaltrials.gov/ct2/show/NCT05162768, accessed on 8 May 2022). Elamepretide was also tried in subjects with Barth Syndrome. A phase 2/3 trial to evaluate safety and efficacy of elamipretide in subjects with Barth syndrome (https://clinicaltrials.gov/ct2/show/NCT03098797, accessed on 8 May 2022) was recently completed, but no final results have been published yet. This study consists of a 12-week crossover phase followed by an open-label extension. Primary endpoints assessed are a 6 min walk test (6MWT) and Barth Syndrome Symptom Assessment (BTHS-SA) Total Fatigue Score. Initial results from 12 individuals showed that elamipretide was well-tolerated but primary endpoints were not met during the 12 weeks crossover phase. After the 36-week open-label phase, the eight remaining subjects showed significant improvements in 6MWT (*p* = 0.02) and BTHS-SA Total Fatigue Score (*p* = 0.03), and there was also a trend toward improved left-ventricular end-diastolic volume [108].

Liver transplant is currently the only treatment option for liver failure in individuals with MPV17 deficiency, but this is still controversial given the multisystem nature of this disorder [93]. Gene therapy was tried in animal models [109]. Given the role of MPV17 in maintaining nucleotide supply in mitochondria, increasing the availability of nucleotide pools could be a potential therapeutic target. In a yeast model with *SYM1* mutation, the ortholog of the human *MPV17*, several molecules were identified that could increase the dTTP pool and mtDNA stability. Interestingly, some of these molecules affect ergosterol levels and therefore could work through increasing the permeability of mitochondrial membranes [110]. There is an ongoing phase 2 clinical trial to evaluate supplementation of deoxycytidine and deoxythymidine in children with mtDNA depletion syndromes (including MPV17 deficiency) (https://clinicaltrials.gov/ct2/show/NCT04802707, accessed on 8 May 2022).

Despite the increased number of preclinical and clinical trials, several challenges are still encountered while conducting such trials for mtDNA maintenance defects. First, these conditions are rare, and recruiting the required number of participants with a particular gene defect remains an obstacle. Including individuals with mtDNA maintenance defects caused by variable genetic defects may allow the recruitment of larger numbers; however, it will affect the homogeneity of the cohort as variable subgroups can have difference responses to the intervention. Furthermore, defects in proteins involved in mtDNA maintenance can be associated with variable phenotypes such as MPV17-related disorders that can have an early-onset severe presentation and late-onset presentation with milder manifestations. Such variability creates challenges even when trials evaluate specific disorders due to single gene defect. Finally, lack of adequate information about the natural history of many mtDNA maintenance defects can make the assessment of the effect of the intervention more difficult. Data sharing, registries, natural history studies, better recognition and diagnosis, and multicenter collaborations can all help towards overcoming many of these challenges.

## 8. Conclusions

In this review, we discussed in detail how mitochondrial membranes interact with the mitochondrial genome and how this interaction is crucial in mtDNA organization and maintenance. MtDNA maintenance defects represent a growing list of disorders, and as we discussed here, several of these disorders result from perturbations in mitochondrial membranes that in turn affect mtDNA replication machinery. Twinkle, TFAM, and ADAT3 are IMM proteins that function in nucleotide organization, which is essential to maintain mtDNA. Defects in these proteins result in variable mtDNA maintenance defects. OPA1, MFN2, DRP1, and MFF are mitochondrial membrane proteins that are involved in mitochondrial fusion and fission. As the dynamic nature of the mitochondria is essential for maintaining a balanced mtDNA replication enzyme, defects in OPA1, MFN2, DRP1, and MFF are also associated with mtDNA maintenance defects. Cardiolipin is an essential lipid in mitochondrial membranes that plays roles in mitochondrial fission and fusion, and also binds to nucleoids; therefore, it is involved in mtDNA maintenance. Deficiency of cardiolipin transacylase, the enzyme that generates mature cardiolipin, affects mtDNA maintenance and causes Barth syndrome. Finally, as mtDNA synthesis requires a constant and balanced supply of dNTPs, the IMM nucleotide transporters MPV17 and ANT1 are essential to maintain mtDNA. Defects in these transports are also associated with different forms of mtDNA maintenance defects.

In spite of our current understanding of mitochondrial physiology and pathophysiologic mechanism of different mitochondrial disorders, still there is a gap of knowledge that hopefully will be filled with advance in current technologies and availability of various animal models. This will help in tailoring more efficient and specific therapies for these debilitating diseases.

## Figures and Tables

**Table 1:** Lists of disorders related to mitochondrial-membrane proteins involved in mitochondrial-genome organization and maintenance.

Mechanism	Gene	Disease	Inheritance
**Nucleoid Organization**	*TFAM*	Mitochondrial DNA depletion syndrome 15 (hepatocerebral type) (MIM#617156)	AR
	*TWNK*	autosomal recessive infantile-onset spinocerebellar ataxia (IOSCA) (MIM# 271245)	AR
		Perrault syndrome (MIM#:616138)	AR
	*ATAD3A*	Harel–Yoon syndrome (MIM#617183)	AR/AD
		Cerebellar hypoplasia, hypotonia, and respiratory insufficiency syndrome, neonatal lethal (MIM#618810)	AR
**Mitochondrial fission and fusion**	*DNM1L*	Encephalopathy, lethal, due to defective mitochondrial peroxisomal fission 1 (MIM#614388)	AR/AD
		Optic atrophy 5 (MIM#610708)	AD
	*MFF*	Encephalopathy due to defective mitochondrial and peroxisomal fission 2 (MIM#617068)	AR
	*OPA1*	Mitochondrial DNA depletion syndrome 14 (encephalocardiomyopathic type) (MIM#616896)	AR
		Behr syndrome (MIM#210000)	AR
		Optic atrophy 1 (MIM#165500)	AD
		Optic atrophy plus syndrome (MIM#125250)	AD
	*MFN2*	Charcot–Marie–Tooth disease, axonal, type 2A2 (MIM# 609260/617087)	AD/AR
		Optic atrophy plus syndrome	AD
**Cardiolipin Remodeling**	*TAFFAZIN*	Barth syndrome (MIM#302060)	XLR
**Mitochondrial membrane transporters**	*MPV17*	Mitochondrial DNA depletion syndrome 6 (hepatocerebral type) (MIM#256810)	AR
		Charcot–Marie–Tooth disease, axonal, type 2EE (MIM#618400)	AR
	*SLC25A4*	Progressive external ophthalmoplegia with mitochondrial DNA deletions, autosomal dominant 2 (MIM#609382)	AD
		Mitochondrial DNA depletion syndrome 12A (cardiomyopathic type) (MIM#617184/615418)	AR/AD
